# Feasibility and safety of CT‐aided pericardiocentesis from a subxiphoid anterior approach by using fluoroscopy in patients with chronic pericardial effusions

**DOI:** 10.1002/clc.23810

**Published:** 2022-03-09

**Authors:** Yu‐Ki Iwasaki, Yuhi Fujimoto, Kanako Ito‐Hagiwara, Eiichiro Oka, Hiroshi Hayashi, Yoshiaki Kubota, Hiroshige Murata, Teppei Yamamoto, Hideki Miyachi, Shuhei Tara, Yukichi Tokita, Kenji Yodogawa, Takeshi Yamamoto, Hitoshi Takano, Wataru Shimizu

**Affiliations:** ^1^ Department of Cardiovascular Medicine Nippon Medical School Tokyo Japan

**Keywords:** fluoroscopic guide, pericardial effusion, pericardiocentesis, ultrasound guide

## Abstract

**Background:**

Pericardiocentesis is an essential procedure for the diagnosis and treatment of pericardial effusions. The purpose of this study was to evaluate the feasibility and safety of a subxiphoid anterior approach using fluoroscopy aided by a sagittal axis chest computed tomography (CT) view in comparison with an ultrasound‐guided apical approach in patients with chronic pericardial effusion.

**Methods:**

Among 72 consecutive patients (68.8 ± 14.4 years old, 52 males) with hemodynamically stable chronic pericardial effusions, a total of 85 procedures were retrospectively analyzed. We divided them into two groups according to the site of the approach for the pericardiocentesis.

**Results:**

A subxiphoid anterior approach (*n* = 53) was performed guided by fluoroscopy. The sagittal axis view of the chest CT was constructed to determine the puncture angle and direction for the subxiphoid anterior approach. An apical approach (*n* = 32) was performed by ultrasound guidance. The success rates of the anterior and apical approaches were 98.1% and 93.8%, respectively. There were two cases with cardiac perforations in the apical approach group, while no cases developed perforations in the subxiphoid anterior approach group.

**Conclusion:**

The subxiphoid anterior approach for pericardiocentesis was feasible and safe for managing chronic pericardial effusions. A reconstruction of the sagittal axis view of the chest CT imaging was helpful to identify the direction and depth to access the pericardial space from the subxiphoid puncture site before the pericardiocentesis using the lateral fluoroscopic view.

## 1 INTRODUCTION

Pericardiocentesis, which is one of the essential procedures for the cardiologist, is applied for the treatment and diagnosis of patients with pericardial effusions. There are several approaches for pericardiocentesis such as an apical approach, parasternal approach, and subxiphoid approach.^1^, ^2^, ^3^, ^4^ The puncture site for the pericardiocentesis is usually decided by ultrasound and depends on the localization of the echo‐free space. The approach to the pericardial space in patients with a hemodynamically stable pericardial effusion is straightforward for experienced physicians. However, it is sometimes technically challenging in patients whose pericardial effusion is distributed more dominantly posteriorly rather than on the anterior or lateral ventricular wall. On the other hand, a fluoroscopic‐guided subxiphoid anterior approach is often chosen for the pericardial access during radiofrequency catheter ablation of ventricular tachyarrhythmias.^5^, ^6^ The lateral right−left fluoroscopic view with a contrast medium injection could identify the anatomical location of the tip of the puncture needle on the anterior aspect of the pericardium. In addition, a reconstruction of the sagittal view along the median line from the conventional axial view of the chest computed tomography (CT), which is often examined to assess underlying disease and the amount and distribution of an effusion in the pericardial space, is an identical image to that in the lateral fluoroscopic view. Therefore, the sagittal view of chest CT imaging before the pericardial puncture might be useful for deciding the subxiphoid puncture site, angle, and distance of the targeted pericardial space. We investigated the feasibility and safety of a CT‐aided subxiphoid anterior approach using fluoroscopy in comparison with an ultrasound‐guided apical approach in the patient with hemodynamically stable pericardial effusions.

## 2 METHODS

**Figure 1:**
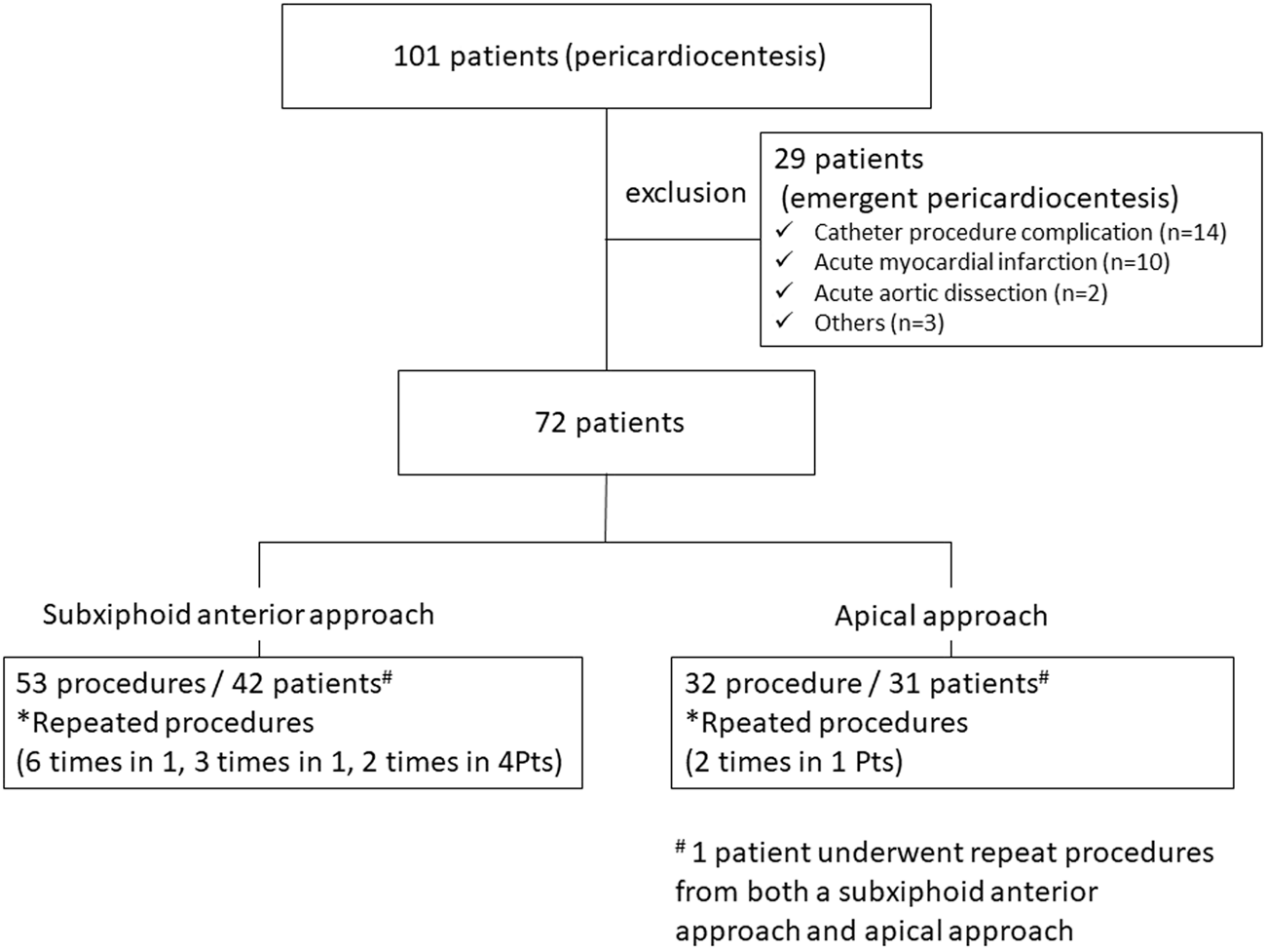
Study population and patient flowchart

One hundred one consecutive patients who underwent pericardiocentesis and pericardial drainage from January 2014 to December 2020 at the Department of Cardiovascular Medicine, Nippon Medical School Hospital were enrolled in the present study. An emergent pericardiocentesis for cardiac tamponade caused by a cardiac perforation associated with catheter ablation procedures or cardiac catheterization and a hemopericardium due to an acute myocardial infarction or acute aortic dissection were excluded from this study (Figure 1). We retrospectively analyzed the remaining 72 patients. The patient characteristics and causes of the pericardial effusions are shown in Table S1. Approximately 56% of the patients had malignant diseases such as lung cancer and breast cancer, and a pericardial effusion was incidentally confirmed by chest CT. The patients were referred to our department of cardiovascular medicine for further evaluation and treatment. Therefore, chest CT was performed within 1 week before the pericardiocentesis in 61 out of 85 cases (82%). Among the 72 patients, 8 required a repeat pericardiocentesis (6 times in 1, 3 times in 1, and 2 times in 6 patients). Therefore, a total of 85 procedures were analyzed. We divided them into two groups according to the approach to the pericardial space. One was the subxiphoid anterior approach (53 procedures) and the other the apical approach (32 procedures). Pericardiocentesis was performed by five physicians in our hospital, and all of the operators were cardiologists. The methods for the pericardiocentesis used, whether a subxiphoid or apical approach was at the discretion of the operator.

The pericardial effusions were diagnosed by transthoracic echocardiography and/or chest CT in our hospital. After written informed consent was obtained, pericardiocentesis was performed in the supine position in the angiography room. The electrocardiogram, oxygen saturation, and noninvasive blood pressure were recorded on a polygraph system (FCL‐2000, Fukuda Denshi). Mild conscious sedation was established by the intravenous administration of midazolam (5−10 mg), pentazocine (15 mg), and hydroxyzine (25 mg). This study was approved by the institutional ethics committee of the Nippon Medical School Hospital (B‐2020‐323).

### 2.1 Subxiphoid anterior approach procedure aided by sagittal axis CT imaging

**Figure 2:**
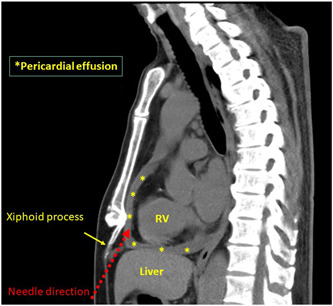
Reconstruction of the sagittal axis view of the chest CT. The Xiphoid process could be a suitable landmark for the subxiphoid anterior approach. The red dotted arrow indicates the needle direction into the pericardial space with a subxiphoid approach. The asterisks indicate the distribution of the pericardial effusion. CT, computed tomography; RV, right ventricle

**Figure 3:**
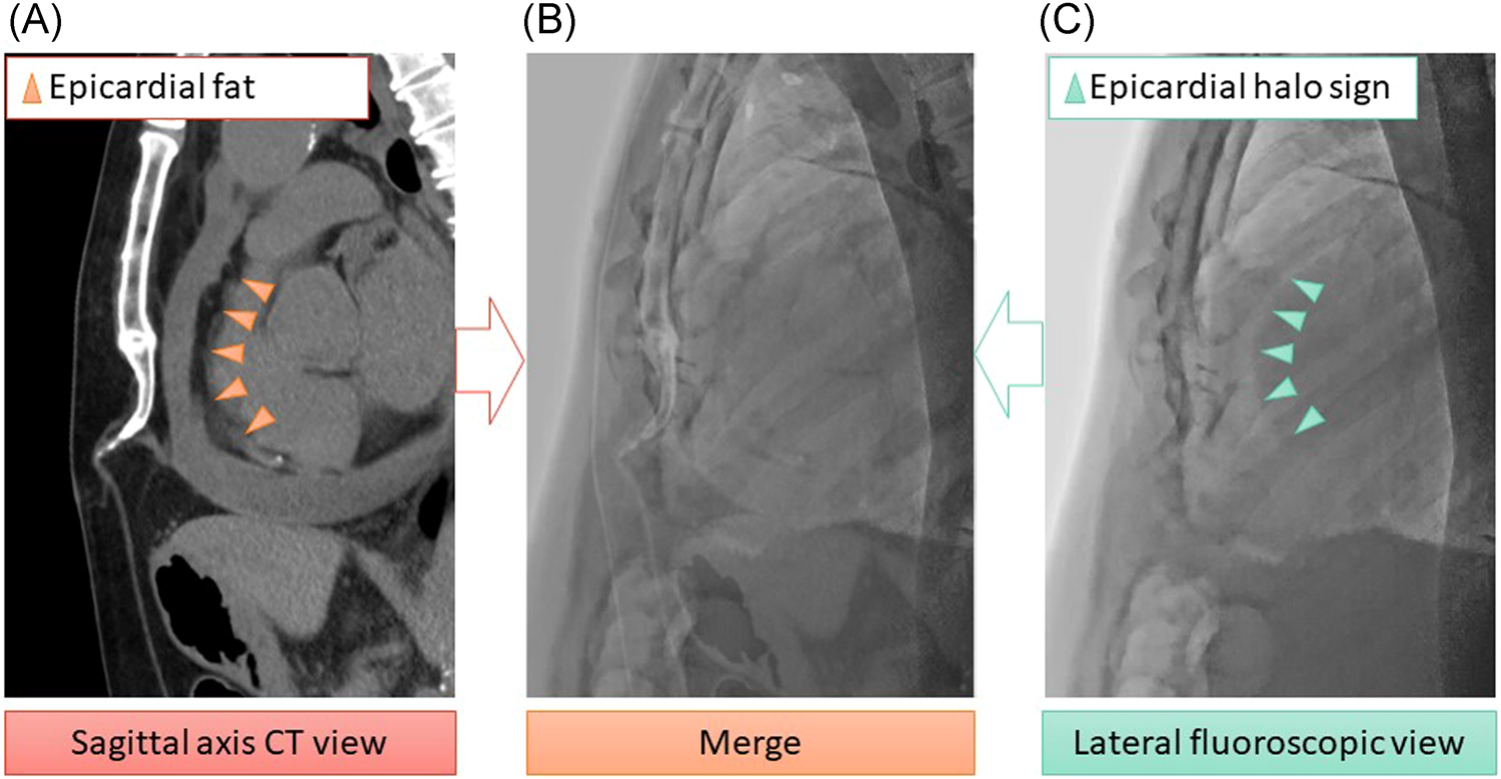
(A) The sagittal axis view along the median line indicates the epicardial fat and a pericardial effusion on the anterior aspect of the heart. (B) A merge view between the sagittal axis CT and lateral fluoroscopic view. (C) The lateral R−L fluoroscopic view exhibits an “epicardial halo sign” in an identical location as the epicardial fat. The anterior aspect of the area with the epicardial halo sign and below the sternum represent the pericardial effusion space. CT, computed tomography

Conventional axial chest CT imaging is useful for the identification of the distribution and amount of a pericardial effusion. There is less information on the needle direction and approach to the pericardial space from a subxiphoid approach. Multiplanar reconstruction CT imaging was performed before the subxiphoid anterior approach procedure. The acquired data from a normal axial plane chest CT image were converted to a sagittal axis view with a 5 mm slice thickness. Anatomical information including the direction and depth to the pericardial space and position of the liver could be obtained by the sagittal axis view CT image along the median line which was identical to fluoroscopic image in the lateral R−L view (Figures 2 and 3). However, the sagittal axis view CT image was not merged with fluoroscopic imaging but was displayed beside the fluoroscopy view during the pericardiocentesis procedure.

**Figure 4:**
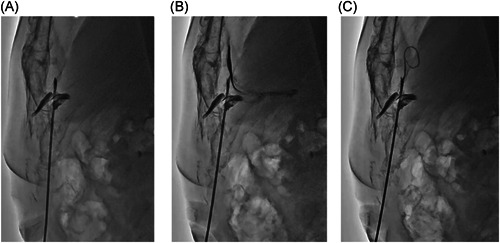
(A) Tenting of the pericardium by the puncture needle was confirmed with an injection of contrast medium. (B) The injection of the contrast medium clearly showed that the tip of the needle was located in the pericardial space. (C) A guidewire was inserted through the puncture needle and placed in the pericardial space.

The puncture site was approximately 3 cm away from the xiphoid process on the caudal side. A puncture from just below the xyphoid process would make the insertion difficult because of the angulation of the puncture needle for the anterior approach. After local anesthesia with 1% lidocaine was established, a skin incision with a surgical scalpel at the puncture site was created and blunt dissection of the subcutaneous tissue by a surgical pean was performed. Manual compression of the subxiphoid puncture site was performed by hand along with a pericardial puncture system including both a needle and syringe containing contrast medium. Therefore, the puncture needle was held almost parallel to the craniocaudal axis. An 18‐gauge puncture needle attached to a 10cc syringe with contrast medium was introduced from the subxiphoid incision almost parallelly (0−15°) to the sternum toward the pericardial space. When the patients who had rich anterior epicardial fat, a lateral fluoroscopic view could identify the beating epicardial fat silhouette, the so‐called “epicardial halo sign” (Figure 3, Supporting Information Movie 1). When the puncture needle reached the anterior side of the pericardium, advancing the needle could drain the pericardial effusion in some of the cases. On the other hand, in the case of a small pericardial effusion on the anterior side, an injection of contrast medium (0.5cc) revealed tissue staining between the pericardium and sternum, and the puncture needle was carefully advanced with a slightly deep angle (15−30°). At that point, too excessive an injection of contrast medium into the anterior mediastinum should be avoided because it would make the visualization difficult due to the contrast medium. After tenting of the pericardium was confirmed (Figure 4A, Supporting Information Movie 2), the needle was advanced into the pericardial space. After contrast medium was confirmed to have been injected into the pericardial space (Figure 4B), a spring guidewire was inserted at least 50 cm into the pericardial space just to make sure that the spring guidewire was surely located within the pericardial space (Figure 4C). The position of the guidewire should be confirmed by several fluoroscopic views including the anterior−posterior (AP) and right anterior oblique (RAO) views (Figure S1). After dilation of the puncture site with a dilator, a pigtail catheter for pericardial drainage was introduced over the spring guidewire.

### 2.2 Apical approach

The apical approach was performed in the supine position on the table in the angiography room. An ultrasound examination was performed to determine the optimal apical puncture site. The puncture site and needle direction for the percutaneous access for pericardial effusion drainage were determined by identifying the site of the greatest separation of the ventricular wall from the parietal pericardium on ultrasound. After local anesthesia with 1% lidocaine was established, an 18G puncture needle was advanced toward the pericardial space. When the pericardial fluid was drained, a guidewire was introduced into the pericardial space guided by fluoroscopy. The guidewire was carefully inserted into the pericardial space, and the guidewire position was confirmed by several fluoroscopic views (i.e., AP, left anterior oblique [LAO], RAO, and R‐L views). After dilation of the puncture site with the dilator, a pigtail drainage catheter was introduced over the guidewire.

### 2.3 Pericardial effusion drainage

After successful placement of the pigtail drainage catheter into the pericardial space, drainage of the pericardial effusion was performed. The amount of the pericardial effusion drainage was limited to approximately 1000 ml per day to avoid pericardial decompression syndrome. The pigtail catheter was removed when the total pericardial fluid was less than 20 ml per day.

### 2.4 Statistical analysis

The data are expressed as the mean ± standard deviation for continuous variables and as absolute frequencies and percentages for categorical variables. For continuous variables and categorical variables, the differences between the groups were compared with a Student's *t *test and Fisher's exact test, respectively. *p *values less than 0.05 were considered statistically significant. All analyses were performed with GraphPad Prism version 8.0 software (GraphPad Software, Inc.).

## 3 RESULTS

The present study showed that pericardial effusions associated with malignancy accounted for 56%, and the majority were lung cancer. Acute pericarditis and collagen disease accounted for 11% and 10% of the cases, respectively. The blood pressure and heart rate at baseline were 131±24/81 ± 16 mmHg and 93 beats per minute, respectively. There were no significant differences in the baseline blood pressure, heart rate, and etiology of the pericardial effusion except for lung cancer (Table S1). Successful pericardial drainage was performed in 82 cases (96.5%). There were no statistically significant differences in the success rate between the two groups (anterior approach: 98.1% vs. apical approach: 93.8%, *p* = .5535). In one case, the pericardial drainage was abandoned because of a pericardial adhesion. An anterior approach in one case was avoided because the reconstructed sagittal image showed no space for a subxiphoid approach due to the position of the liver. Successful pericardiocentesis was performed from an apical approach instead of a subxiphoid approach.

There were two cases of misplacement of the pigtail drainage catheter into the ventricle, which required a surgical repair of the perforation site. Both cases were in the apical approach group and the perforation sites were on the right ventricular free wall and left ventricular apex. None of the patients developed a pneumothorax, liver injury, or colon injury. Among the 82 successful cases, the total drainage volume of the initial pericardiocentesis was 591 ± 297 ml. There were no significant differences in the drainage volume between the subxiphoid and apical approaches (subxiphoid: 619 ml vs. apical approach: 555 ml, *p* = .341). Overall, the heart rate and diastolic pressure had significantly decreased after the pericardial drainage. There were no significant differences in the hemodynamic parameters between the subxiphoid and apical approaches (Table S2).

## 4 DISCUSSION

The present study confirms the feasibility and safety of a CT‐aided pericardiocentesis from a subxiphoid anterior approach using fluoroscopy in patients with a hemodynamically stable pericardial effusion. A multiplanar reconstruction in the sagittal view image helps determine the appropriate approach site, puncture angle, and depth to access the pericardial space for the subxiphoid anterior approach before the pericardiocentesis, leading to a high success rate and low complication rate.

### 4.1 Pericardiocentesis for chronic pericardial effusions

The pericardial space usually contains a small amount of the pericardial fluid.^7^ Various pathophysiological conditions such as inflammation, malignancy, and collagen disease increase the production of pericardial fluid. A rapidly developing pericardial effusion such as a free wall rupture associated with an acute myocardial infarction and catheter perforation during catheter ablation procedures increases the intrapericardial pressure leading to cardiac tamponade. On the other hand, a slowly developing pericardial effusion does not affect the hemodynamics and results in a large amount of pericardial fluid. Recently, the prevalence of malignant disease is on the rise and chemotherapy and radiation therapy may cause a pericardial effusion.^8^, ^9^ Chronic pericardial effusions associated with malignancy are more likely to develop into cardiac tamponade as compared with those of other etiologies.^10^ The evaluation of a pericardial effusion helps in the differential diagnosis of the cause of the pericardial effusion along with an improvement in the hemodynamic condition and clinical symptoms. Therefore, the opportunity for pericardiocentesis for both the diagnosis and treatment will increase for physicians (especially for cardiologists). Complications of elective pericardiocentesis procedures in patients with chronic pericardial effusions should be avoided especially in patients with a systemically diseased condition such as a malignancy.

In addition to the fluoroscopic‐guided approach, there are two modalities for pericardiocentesis. An ultrasound‐guided approach for the pericardiocentesis has been developed as a standard technique both for elective and emergent cases. It can be feasible from a parasternal,^2^, ^11^ subxiphoid,^12^, ^13^ or apical site^1^ by using ultrasound. In general, the approach to the pericardial space is decided by the distribution of the pericardial effusion and the operator's preference. The puncture site should be chosen by the distribution of the pericardial effusion. The feasibility and safety of a real‐time CT‐guided needle puncture for pericardiocentesis has been reported.^14^ The operator relies on the CT acquisitions for the needle puncture to access the pericardial space. The puncture can be performed from a ventrolateral entry site. In our hospital, the ultrasound‐guided apical and fluoroscopic‐guided subxiphoid anterior approaches were usually chosen in the angiography room for an elective pericardiocentesis.

### 4.2 Benefits of a subxiphoid anterior approach

There are several benefits of a subxiphoid anterior approach. The inferior and apical regions of the ventricle wall move in the caudal direction in synchrony with the respirations. The dynamic wall motion of the heart and lungs might increase the risk of a cardiac perforation or a pneumothorax during the apical approach. Contrary to that, the motion of the anterior aspect of the pericardium associated with respirations was limited as shown in Supporting Information Movie 2. The direction of the puncture needle had a shallow angle with the surface of the anterior aspect of the pericardium, which could reduce the risk of cardiac perforations during the beating heart wall motion. Therefore, the operator concentrated on the procedure of advancing the needle regardless of the respirations when using the subxiphoid anterior approach.

Recently, the subxiphoid anterior approach as well as the subxiphoid posterior approach have been chosen as a standard pericardial approach for catheter ablation of ventricular tachyarrhythmias.^5^, ^15^ A successful pericardial approach using this method was achieved in spite of the fact that there was only a physiological amount of pericardial fluid in those cases. Therefore, the anterior approach could apply to pericardiocentesis in patients with chronic pericardial effusions.

### 4.3 Reconstruction of the chest CT imaging in the sagittal axis view

Several modalities including ultrasound, CT, and magnetic resonance imaging are chosen for the assessment of pericardial effusions to determine the amount and distribution of the effusion in the pericardial space. Ultrasound is a gold standard modality for the diagnosis and assessment of pericardial effusions. In addition, chest CT is also useful for evaluating the detailed distribution and amount of the pericardial effusion and its cause. In the present study, the majority of the patients underwent a chest CT before the pericardiocentesis to diagnose any underlying disease and assess the pericardial effusion. Reconstruction of the sagittal axis view helped us determine the anatomical information for the direction and distance of the needle puncture without a time‐consuming process and additional radiation exposure. Moreover, the sagittal axis view along the median line was identical to the fluoroscopic lateral R−L view, which could identify an epicardial halo sign indicating epicardial fat.^16^ The present study revealed that the epicardial halo sign in the R−L fluoroscopic view was identical to the epicardial fat in the chest CT sagittal view (Supporting Information Movie 1). While referencing the sagittal axis view along the median line of the chest CT before the procedure, the operator was able to imagine the pericardiocentesis procedure in advance. A real‐time fluoroscopic lateral view could visualize the position of the puncture needle as well as the epicardial fat. The pericardial space was verified with an injection of a very small amount of contrast medium. As long as the contrast medium pooled in the mediastinal fat, the needle did not reach the pericardial space.

### 4.4 Disadvantages of the subxiphoid anterior approach

The pericardiocentesis procedure should be performed in an angiography room, which requires more time and medical staff. There is a risk of radiation exposure during the procedure as compared with that of an ultrasound‐guided apical approach. The subxiphoid anterior approach was feasible for the pericardiocentesis in the majority of the patients. However, it was difficult to access the pericardial space in patients whose liver was located just below the xiphoid process. A sagittal axis reconstruction of the chest CT before the procedure was useful to identify the location of the liver and xyphoid process. In that case, the apical approach should be chosen instead of a subxiphoid approach. Liver injury associated with pericardiocentesis has been reported especially with a subxiphoid inferior approach.^3^, ^4^

### 4.5 Complications of pericardiocentesis

There are several complications associated with pericardiocentesis including cardiac perforation, coronary injury, pneumothorax, hemothorax, liver injury, and colon injury.^3^, ^4^, ^17^ It has been reported that the cardiac perforation rate is ≈1% and life‐threatening direct complications associated with cardiac perforations are less than 1%.^18^, ^19^ In the present study, cardiac perforations developed in 2 out of 86 procedures (2.3%) and both cases required a surgical repair, and that was a higher incidence than in the previous reports. The reason for the different rates of complications was unclear but it might have been associated with the small study population. However, efforts to reduce the complication rate of pericardiocentesis especially in elective procedures should be given attention. It is important to confirm that the contrast medium injected from the tip of the needle actually reaches the pericardial space, even after the advancement of the guidewire has been confirmed to surely be located within the pericardial space and not within the ventricle or great vessels. The puncture site dilatation and drainage catheter insertion through a wrongly positioned guidewire might increase the complications that might require a surgical repair by a thoracotomy. Several views, such as the LAO and RAO views in addition to a lateral fluoroscopic view, were useful for the identification of the guidewire before inserting the drainage catheter. If the guidewire was advanced beyond the cardiac silhouette, the position of the guidewire might have been located in the pulmonary artery/vein or a great vessel through the ventricle or pleural space. In that case, the guidewire should be carefully removed and would require close monitoring of the amount of the pericardial effusion as well as the vital signs. Even for identifying an incorrect puncture, only a misplaced guidewire usually can be removed without the development of serious complications other than a coronary artery perforation.

In the case of adequate echo‐free space at the puncture site, an ultrasound‐guided apical approach is safe and useful for patients with chronic pericardial effusions. However, the pitfall of the ultrasound‐guided approach is that there is no real‐time visualization during the needle puncture. In addition, several attempts of the needle puncture from the apex might cause a fluid shift from the pericardial space to the pleural space resulting in a less safe pericardial space margin at the apex. If the echo‐free space in the apical view is not adequate for a safe pericardial puncture, the subxiphoid anterior approach should be considered instead.

It has been reported that after rapid drainage of a large volume, pericardial decompression syndrome can develop, which causes acute heart failure and pulmonary edema after pericardial drainage.^20^ In the present study, there were no cases with pericardial decompression syndrome presumably because of the limited drainage of up to 1000 ml per day in principle. The average drainage volume was 591 ml. The incidence of pericardial decompression syndrome is rare, but it is associated with significant mortality. It is important to limit the first drainage volume and continue drainage with a slow rate from a pigtail catheter.

### 4.6 Application for emergent cases requiring pericardiocentesis

The present study focused on pericardiocentesis in patients with hemodynamically stable pericardial effusions. All of the procedures were performed under elective conditions in the angiography room. Emergent cases such as cardiac tamponade associated with cardiac catheterization or catheter ablation were excluded. However, the subxiphoid anterior approach can be applied for the treatment of cardiac tamponade associated with iatrogenic cardiac perforations. The incidence of cardiac tamponade during catheter ablation and percutaneous coronary intervention for chronic total occlusions has been reported to be 0.2−5%.^21^, ^22^, ^23^ The complication rate is not so high, but the opportunity will become higher along with the increase in the volume of catheter ablation procedures for AF and percutaneous catheter intervention procedures. Immediate pericardial drainage will be needed to bail out hemodynamically collapsed situations. In such a situation, there is not an adequate echo‐free space in the case of an acute pericardial effusion leading to cardiac tamponade. It also takes time to prepare a working space for an ultrasound‐guided pericardiocentesis during cardiac catheterization. A fluoroscopic‐guided subxiphoid anterior approach requires only sterilization of the puncture site without any preparation. Moreover, the procedure technique and workflow are completely the same and skilled physicians could perform a fluoroscopic‐guided pericardiocentesis without CT imaging. Gaining experience with the subxiphoid anterior approach for chronic pericardial effusions will be helpful even for performing even emergency cases.

### 4.7 Limitations

Firstly, the present study was a single‐center experience with a small study population. The patient background and etiology of the pericardial effusions differed in each hospital. Therefore, the results might not be directly applied to the general clinical practice. The distribution and location of the pericardial effusions were diverse and an optimal approach for the pericardiocentesis should be decided for individual patients. However, a pericardiocentesis using a subxiphoid anterior approach in patients with chronic pericardial effusions is one of the important technical methods for a safe procedure in some cases. Secondly, an anterior approach in patients with suspected pericardial adhesions to the anterior aspect of the heart due to a prior history of pericarditis or cardiac surgery is not suitable for this technique, which could result in the risk of cardiac perforation. A pre‐evaluation by chest CT might be useful in these cases. Thirdly, the study design had a retrospective nature implying the operator's preference might have affected the study results including the success rate and complication rates. A large‐scale multicenter study will be needed to clarify these issues.

## 5 CONCLUSION

Fluoroscopic‐guided pericardiocentesis from a subxiphoid anterior approach was feasible and safe for managing patients with hemodynamically stable pericardial effusions. A multiplanar reconstruction of the chest CT images in the sagittal view along the median line was helpful for performing the pericardiocentesis using the lateral fluoroscopic view.

## CONFLICTS OF INTEREST

The authors declare no conflicts of interest.

## Supporting information

Pericardiocentesis from subxiphoid anterior approach.

Supporting information.

Supporting information.

## Data Availability

The data that support the findings of this study are available from the corresponding author upon reasonable request.
