# Supplementary figures and images for: Feasibility and safety of CT‐aided pericardiocentesis from a subxiphoid anterior approach by using fluoroscopy in patients with chronic pericardial effusions
Source: Clin Cardiol. 2022 Mar 9;45(5):519–26. doi: 10.1002/clc.23810 (PMC9045084; doi:10.1002/clc.23810)

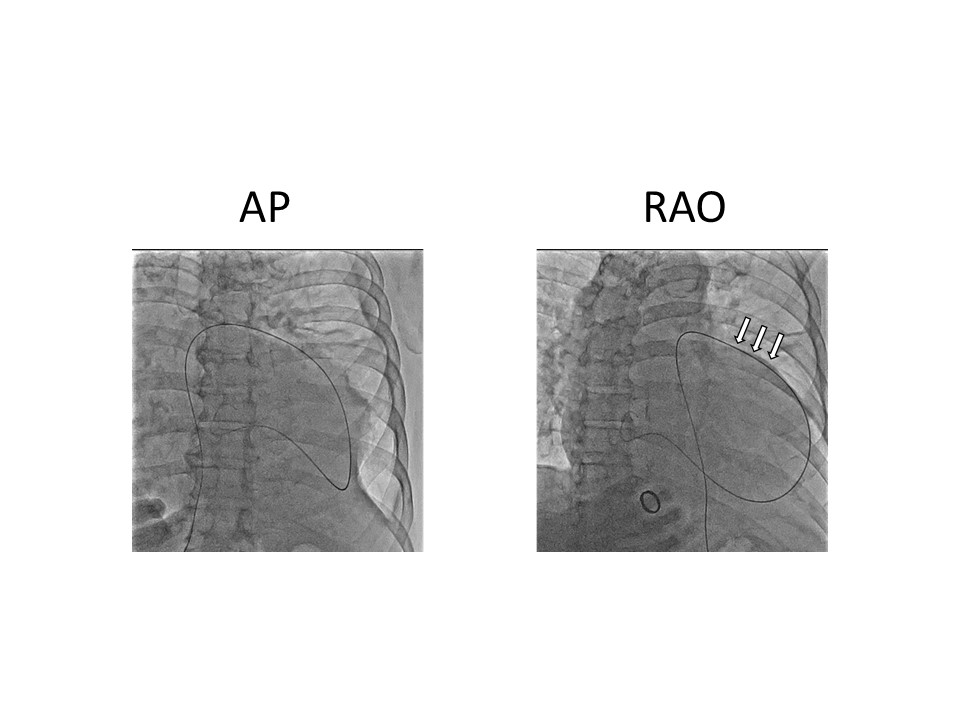

Supplement: Supplementary file 3 — Supporting information. [file CLC-45-519-s002.jpg]
